# Combining Multicolor FISH with Fluorescence Lifetime Imaging for Chromosomal Identification and Chromosomal Sub Structure Investigation

**DOI:** 10.3389/fmolb.2021.631774

**Published:** 2021-03-17

**Authors:** Archana Bhartiya, Ian Robinson, Mohammed Yusuf, Stanley W. Botchway

**Affiliations:** ^1^ London Centre for Nanotechnology, University College London, London, United Kingdom; ^2^ Research Complex at Harwell Rutherford Appleton Laboratory, Didcot, United Kingdom; ^3^ Condensed Matter Physics and Materials Science Division, Brookhaven National Lab, Upton, NY, United States; ^4^ Centre for Regenerative Medicine and Stem Cell Research, Aga Khan University, Karachi, Pakistan; ^5^ Central Laser Facility, Science and Technology Facilities Council, Rutherford Appleton Laboratory, Oxon, United Kingdom

**Keywords:** chromosome, fluorescence lifetime imaging, multicolor FISH, microscopy, multiphoton, karyotyping

## Abstract

Understanding the structure of chromatin in chromosomes during normal and diseased state of cells is still one of the key challenges in structural biology. Using DAPI staining alone together with Fluorescence lifetime imaging (FLIM), the environment of chromatin in chromosomes can be explored. Fluorescence lifetime can be used to probe the environment of a fluorophore such as energy transfer, pH and viscosity. Multicolor FISH (M-FISH) is a technique that allows individual chromosome identification, classification as well as assessment of the entire genome. Here we describe a combined approach using DAPI as a DNA environment sensor together with FLIM and M-FISH to understand the nanometer structure of all 46 chromosomes in the nucleus covering the entire human genome at the single cell level. Upon DAPI binding to DNA minor groove followed by fluorescence lifetime measurement and imaging by multiphoton excitation, structural differences in the chromosomes can be studied and observed. This manuscript provides a blow by blow account of the protocol required to perform M-FISH-FLIM of whole chromosomes.

## Introduction

Nuclear chromosomes are composed of chromatin, primarily a complex of deoxyribonucleic acid (DNA) and proteins, which are arranged into elementary structural units of nucleosomes, each made up of 147 base pairs. The nucleosome is composed of eight core histone proteins, in a bead on a string structure forming 11 nm fibers with DNA wrapped around the nucleosomes. Further coiling of these units leads to highly compact structures. The organization of chromatin into these higher-order structures and how they are controlled play a role in the so-called DNA condensation process which still remain a subject of debate as well as representing one of the key challenges in structural biology. Traditional methods for identifying chromosomes based on large scale (> 100 nm) structure include Giemsa banding (G-banding) method that displays chromosomes having dark (AT rich regions) and light (GC rich regions) band, heterochromatin and euchromatin respectively (Sumner, 1982). This method is still currently the gold standard for clinical hospital laboratories and involves analysis at the microscope stage and requires highly specialized training (Liehr, 2017). More recently, a modern and user-friendly Fluorescence *in situ* Hybridization (FISH)-based protocols for karyotyping include Multiplex FISH (M-FISH) is being explored. M-FISH is a fluorescence technique that uses computer-generated colors from a coding scheme, which analyses the fluorescence from various pairs of five paints (probes) and uses DAPI as a counterstain (Speicher et al., 1996). Although M-FISH is an invaluable and powerful karyotyping tool that provides detailed analysis of structural rearrangements and marker chromosomes and does not require intensive training (Yusuf et al., 2013), it lacks detailed information at the sub 500 nm level. Its use has been expanded into validating and karyotyping complex clinical cases after G-banding (Jalal and Law, 1999) on the same sample. Furthermore, its use has been demonstrated well in identifying and karyotyping chromosomes after contrast (Shemilt et al., 2015) and fluorescence (Yusuf et al., 2011) imaging methods. Fluorescence lifetime imaging microscopy (FLIM) is a technique that can map the spatial nature of excited state lifetime and can act as a reporter when combined with a sensor to changes in the fluorophore’s environment (Anthony et al., 2009; Ahmed et al., 2019). FLIM can be generally applied to a broad field of research including viruses, plants, mammalian and material research to name a few (Danquah, et al., 2012; Breeze et al., 2016; Ahmed et al., 2020). The fluorescence lifetime of a molecule in a particular environment is fixed and defines the average time the molecule spends in the excited state before returning to the ground state, which is typically in the range of several nanoseconds (ns). Changes in the measured excited state life values is a characteristic of the fluorophore’s environment such as pH, viscosity, proximity to other molecules and energy transfer events via dipole-dipole interactions. FLIM allows the excited state lifetime of a sample to be measured and imaged on a pixel by pixel basis. FLIM may be constructed around epifluorescence microscope configuration, confocal single and multiphoton excitation microscopy together with intensified charged coupled device (iCCD) or time-correlated single photon counting (TCSPC). Epifluorescence-FLIM mostly use the iCCD method, although recently a wide array single-photon avalanche photo-diode has been demonstrated) (Suhling et al., 2019). The application of frequency-domain lifetime imaging has also been described (Lakowicz, 2006; Schoberer and Botchway, 2014). However, confocal and multiphoton FLIM via TCSPC currently provide the best special and temporal resolution. Although the operation of the different systems varies, the data interpretation is similar. The principle of a TCSPC FLIM system is shown in Schematic 1 (and also detailed in Botchway et al., 2015; Schoberer and Botchway, 2014). FLIM (or the excited state lifetime value) is less influenced by molecule concentration and photo-bleaching whilst providing another method of image contrast. Although the lower the concentration, the longer is the data collection time. Generally, when photo-bleaching is observed, this can be easily accounted for by a Stern-Volmer equation.

Recently, chromosome compaction at nanometer length scales has been suggested along the length of chromosomes imaged using multiphoton FLIM after DAPI staining (Estandarte et al., 2016) alone. This was performed on a classical spread of chromosomes whereby excited state lifetime measurements have been obtained on all 46 chromosomes. Crucial for analyzing the FLIM data is the identification of each chromosome whereby M-FISH plays an important role. Here we describe a step by step procedure combining FLIM together with DAPI staining acting as an environment sensor for investigating chromosome substructure and M-FISH for chromosome identification and karyotyping.

## Materials

All solutions must be prepared using Milli-Q^®^ Millipore water (prepared by purifying deionized water, to attain a sensitivity of 18 MΩ -cm at 25°C).

### Consumable for Extraction of Primary T-Lymphocytes

(1) 10 ml of blood obtained from a human donor.
(2) BD Vacutainer^®^ lithium heparin tubes (Becton Dickinson).
(3) 5 ml of Histopaque-1077 (Sigma-Aldrich).
(4) Hank’s Balanced Salt Solution (HBSS, Life Technologies)
(5) Sterile pastette (Alpha Laboratories, Eastleigh, United Kingdom).

### Requirements for T-Lymphocytes Cell Culture

(1) A sterile cryovial containing 3 × 10^6^ T-lymphocytes cells.
(2) Lymphoblastoid cell growth medium (LCGM): consisting of Roswell Park Memorial Institute [RPMI-1640 (1X)] medium supplemented with 20% heat inactivated fetal bovine serum (FBS), 1 mM sodium pyruvate, 2 mM L-glutamine, 100 U/ml penicillin, 100 μg/ml streptomycin (all from Invitrogen/Life Technologies).
(3) Stimulating growth medium (SR10): comprised of RPMI 1640 supplemented with 10% heat inactivated FBS, 1 mM sodium pyruvate, 2 mM L-glutamine, 100 U/ml penicillin, 100 μg/ml streptomycin, 50 μM, 2-mercaptoethanol (GIBCO/Life Technologies), 20 U/ml recombinant interleukin-2 (IL2; Sigma-Aldrich) and 0.4 μg/ml phytohaemagglutinin (PHA; Sigma-Aldrich).
(4) Growth medium (GR10) comprised of SR10 without PHA.
(5) Lethally irradiated lymphoblastoid GM 1899A as feeder cells.
(6) Incubator at 37°C temperature with 5% C0_2_ supply.
(7) Freeze mix: 10% dimethyl sulfoxide (DMSO, Sigma-Aldrich Company Ltd., Gillingham, United Kingdom) and 90% of FBS (Life Technologies).

### Reagents for Chromosome Preparation

(1) Colcemid (Karyomax, Gibco by Life technologies (10 μg/ml): to arrest the cells at metaphase stage.
(2) KCL (75 mM) to lyse the cells (Sigma-Aldrich).
(3) Methanol: acetic acid at the ratio of 3:1 (v/v) for fixation.
(4) Vectashield mounting medium containing 4′,6-Diamidine-2′-phenylindole dihydrochloride stain (5 μg/ml) DAPI, Victor Laboratories, H-1200).
(5) DAPI (4 μM) (ThermoFisher Scientific) in Milli-Q^®^ Millipore water from stock of 40 μM DAPI.

### Equipment

(1) Counting cells: The ADAM or equivalent cell counter (Labtech International Ltd., Uckfield, United Kingdom), 20 µl of cell media of a stimulated T-cells and 20 µl of each accuStain T and N solutions for total cell count with a propidium iodide dye and non-viable cells count with fluorescent dye respectively.
(2) Epifluorescence microscope (Zeiss Z2Axioimager or equivalent) with 6 filters such as DAPI (counterstain, SP-100), aqua (31036v2), green (MF-101, orange (31003), red (SP-107 (SP-103v1) and near infrared (SP-104v2).
(3) ISIS imaging software (MetaSystems) for capturing images and analysis.
(4) Excitation sources for the multiphoton FLIM were from a Mira 900 F (Ti-sapphire laser (Coherent Ltd., United Kingdom, Tunable 700–980 nm, pulse length 180–200 fs) pumped by a Verdi V18, operating at 532 nm with a CW outputs. Here the laser was tuned to 760 nm. Photons were detected by a hybrid detector HPM 100–40, connected to a time correlated single photon counting PC module, SPC830, Becker and Hickl, Germany.

### Solutions for M-FISH

(1) 0.1× Saline-sodium citrate buffer (SSC) stock: Add 1 ml of 20 x SSC (Sigma-Aldrich) in a 200 ml Milli-Q^®^ Millipore water, pH 7.25. Transfer ∼50 ml of solution in a two Coplin jars, keeping one jar at room temperature and another in a fridge at 4 C.
(2) Repeat the above procedure to prepare 2× SSC stock: Add 20 ml of 20× SSC in a 200 ml Milli-Q^®^ water, pH 7.45. Pour ∼50 ml of solution in a two Coplin jars, keep one jar at 70 C (±1°C) in a hot water bath and another in a fridge at 4 C.
(3) Prepare 0.07 mol/L: add 1 ml of 7 M stock solution in a 100 ml of Milli-Q^®^ water and transfer ∼50 ml of solution in Coplin jar and stored at room temperature.
(4) 100, 95 and 70% of ethanol series prepared in a Milli-Q^®^ water. Keep at room temperature.
(5) 0.4× SSC: mix 1 ml of 20× SSC in a 50 ml of Milli-Q^®^ water, pH 7.2. Keep at 72 C (± 1°C).
(6) 2× SSCT: dissolving 0.05% Tween-20 (Polyoxyethylenesorbitan-monolaurate syrup, sigma P-1379) in a 50 ml of a 2× SSC, pH 7.45 and keep at room temperature.

## Methods

### Cell Culture

#### Lymphoblastoid Cell Line Culture

(1) Thaw a cryovial containing 3 × 10^6^ cells at 37°C in a water bath for 2 min (mins).
(2) Transfer contents into a 15 ml conical bottom tube containing 10 ml of lymphoblastoid cell growth medium (LCGM). Mix the cells by inverting the tube and centrifuged for 5 min at 1200 rpm (revolution per minute). Remove the supernatant followed by resuspending the cell pellet in 10 ml of LCGM.
(3) Transfer the suspended cells into a vented 25 cm^2^ culturing flask.
(4) Incubate the flask with cells, in the upright position for three days. At this stage, cells become disaggregated. Count the cells at daily intervals using the Adam cell counter [*Equipment* (1)].
(5) Check if the cell number has reached 0.8 × 10^6^, then add 10 ml of fresh LCGM and transfer the cells to 75 cm^2^ flask.

#### Feeder Cell Preparation

(1) Lethally irradiated (40 Gy) lymphoblastoid GM1899A cells are used as feeder cells for human T-lymphocytes (see 4 below).
(2) The culturing conditions for this lymphoblastoid cell line are described in the Section *Lymphoblastoid Cell Line Culture*.
(3) Prepare feeder cells as follows: when cell number reaches 5 × 10^7^ cells per ml, transfer the cells into one or two, 50 ml conical bottom tubes (depending on the volume) and centrifuge at room temperature for 5 min at 1200 rpm. Aspirate the supernatant and resuspend the cell pellet in 5 ml of LCGM.
(4) Lethally irradiate the cells at room temperature with an X-ray source at dose of 40 Gy (Gray), at dose rate 1.7 Gy per min.
(5) Dilute the lethally irradiated feeder cells in the freeze mix [see *Requirements for T-lymphocytes cell Culture*. (7)], Aliquot 1 ml of mixture (cells in 1 ml freeze mix) in each cryovials and freeze at –80 C in a Mr. Frosty™ (Thermo Scientific) containers which allow cooling at rate of 1 C per min. After 24 h, vials are transferred to the liquid nitrogen container for long term storage.

#### Extraction of Human Stimulated T-Lymphocytes

(1) Collect 10 ml of blood from a donor (22 years old female was used in our case) into BD Vacutainer^®^ lithium heparin tubes.
(2) Aliquot 5 ml of Histopaque-1077 into a four ∼15 ml conical bottom centrifuge tubes at room temperature. Mix 10 ml of blood with 10 ml of Hank’s Balanced Salt Solution at a room temperature in ∼50 ml tube.
(3) Layer ∼5 ml of diluted blood slowly onto each of the four tubes containing Histopaque-1077 using a sterile pastette. Centrifuge the tubes at 1600 rpm for 20 min.
(4) Aspirate the top serum layer after phase separation from each tube leaving around 0.5 cm of liquid above the buffy coat cell layer. Collect the buffy coat using a sterile pastette and transfer into a 15 ml tube containing ∼10 ml of HBSS.
(5) After mixing by inverting the tubes, centrifuge at 1200 rpm for 5 min at room temperature. Aspirate the supernatant and re-suspend the cell pellet with ∼5 ml of HBSS.
(6) Centrifuge the cell suspensions at 1200 rpm for 5 min at room temperature. Aspirate the supernatant and wash the cells with ∼10 ml of HBSS once. Perform cell counting using 20 µl aliquot of cell suspension. Centrifuge the tubes at 1200 rpm for 5 min at room temperature and aspirate the supernatant.
(6) Next re-suspend the cells at concentration of 3 × 10^6^ cells per ml in freeze mix.
(7) Freeze cells at –80 C and transfer the cryogenic vials in Mr. Frosty™ container. The next day transfer vials to liquid nitrogen container for long-term storage.

#### Culturing Conditions of T-Lymphocytes

(1) Thaw cryovial containing 3 × 10^6^ cells at 37 C in a water bath for 2 min.
(2) Transfer cells with sterile pastette into a 15 ml conical bottom tube containing 10 ml of stimulating growth medium.
(3) Mix the cells by inverting the tube and centrifuge for 5 min at 1200 rpm, room temperature.
(4) Aspirate the supernatant and re-suspend the cell pellet in 10 ml of SR10 and centrifuge for 5 min at 1200 rpm, room temperature.
(5) In the meantime, thaw a cryovial of feeder cells in the water bath at 37°C for 2 min.
(6) Aspirate the supernatant from T-lymphocytes and re-suspend the cells in 10 ml of SR10.
(7) Transfer the feeder cells with sterile pastette into a 15 ml conical bottom tube containing T-lymphocytes, mix the tube by inverting several times and centrifuge at room temperature for 5 min at 1200 rpm. Aspirate the supernatant and again re-suspend the cell pellet in 10 ml of SR10.
(8) Transfer the cell suspension into a vented 25 cm^2^ flask and incubate at 37°C with 5% CO_2_ at an angle of about 10° from horizontal position.
(9) Leave in incubator for 4 days to grow.
(10) Disaggregate cells and count cells daily.
(11) When cells reached a density of 0.8 × 10^5^ cells per ml, perform a dilution of 1:2 with growth medium.

### Cell Counting and Viability

(1) Use the ADAM cell counter or equivalent for cell counting and viability.
(2) For every cell count, aliquot 20 µl of each “T” and “N” solutions in two separate Eppendorf. Disaggregate the cells by shaking vigorously. Aliquot 20 µl of cell media and mix with T and N solutions separately and incubate for 2 min at room temperature. After 2 min of incubation, load the samples into an appropriate T and N positions in the cartridge and load into the ADAM.
(3) The cell counter provides a value for total cell number (T), viable cell number (N) and percentage of viable cells.

### Chromosome Preparation

(1) Arrest chromosomes at the mitotic stage when the cells are confluent up to 75–80%.
(2) Transfer the cell media from the culturing flasks into a 50 ml falcon tube and centrifuge at 1000 rpm for 10 min.
(3) In the meantime, use 20 µl of cell media to count the cells using an ADAM cell counter. The cell count ranged between 0.32–0.35 × 10^6^ cells per ml.
(4) Aspirate the supernatant and then slowly add 6 ml of pre-warmed (37°C), hypotonic KCL solution (75 mM), in a falcon tube.
(5) Immediately, transfer the tube to 37°C water bath for 8–10 min and then centrifuge at 1000 rpm for 10 min.
(6) Meanwhile, prepare fresh fixative of methanol: acetic acid solution (MAA) also known as carnoys solution at the ratio of 3:1.
(7) Aspirate the supernatant and quickly add carnoys solution in the tube, centrifuge the tube at 1000 rpm for 10 min. Repeat the washing procedure with carnoys solution three washes.
(8) Store the prepared chromosome solutions in –20°C for future use.

### Chromosome Mounting

(1) Clean glass slides (Supersoft, VWR international) by soaking them overnight in 70% ethanol: water solution which effectively removes grease.
(2) Wipe the slides with colourless soft tissues and place in the freezer for 30 min before use.
(3) To prepare chromosome spreads, retrieve the cleaned frozen slides from the freezer, blow on the slides to humidify it and then drop 20 μl–30 μl carnoys fixed chromosomes from a height of around 30 cm to obtain good spreads of chromosomes on the slides.
(4) Place the slides on the hot plate (45°C) to dry. Once the slides are dried, stain with DAPI/antifade (5 μg/ml) then covered with a 22 × 50 mm^2^ cover slip.
(5) Incubate the slides with DAPI for 10 min then observe using an epifluorescence microscope.

### Sample Preparation for Multiphoton Fluorescence Lifetime Imaging Microscopy (FLIM)

(1) For lifetime measurement and FLIM of chromosomes, prepare chromosome spreads according to the sections *Chromosome Preparation* and *Chromosome Mounting*.
(2) Stain fixed chromosomes using 4 μM DAPI and incubate for 20 min in the dark.
(3) Following incubation, soak slides in 1× phosphate buffered saline (PBS, pH-7.4) for 4 min.
(4) Before imaging, mount DAPI stained slides with 1 drop of water and cover with a cover slip (22 × 50 mm^2^, No. 1 or 1.5 depending on the cover glass thickness specifications of the imaging objective).

### Procedure for DNA Hybridization

(1) Prepare 0.1× SSC and 0.2× SSC, pour into Coplin jars and place into the refrigerator at 4°C.
(2) Prepare 2× SSC, pour into a Coplin jar and place into a water bath at 70°C (±1°C).
(3) Carefully remove coverslip from the glass slide following the FLIM data acquisition.
(4) Place slide in a series of ethanol: 70, 95, and 100% for 30 s in each solution and leave to dry in air.
(5) Incubate the slides for 30 min in the prewarmed 2× SSC at 70°C (±±1°C) Coplin jar.
(6) Remove the Coplin jar from the water bath and let it cool for 20 min at room temperature.
(7) Meanwhile prepare the M-FISH probe cocktail according to the intended area for hybridization e.g., 9 µl for an 18 × 18 mm^2^ cover slip, 12 µl for a 22 × 22 mm^2^ cover slip.
(8) Denature the probe by incubating at 75°C (±1°C) for 5 min on the prewarmed hot plate.
(9) Place on ice for 15 s.
(10)Incubate at 37 C (±1°C) for 30 min on the prewarmed hot plate.
(11) Once the slides have cooled to room temperature, place the slides in the Coplin jar containing 0.1× SSC at room temperature for 1 min.
(12) Denature slides in 0.07 N NaoH at room temperature for 1 min.
(13) Place slides into 0.1× SSC 4°C for 1 min.
(14) Place slides into 2× SSC 4°C for 1 min.
(15) Transfer to a Coplin jar with 70% ethanol for 1 min.
(16) Transfer to a Coplin jar with 95 and 100% ethanol and incubate for 1 min. Allow the slides to air dry.
(17) Pipette the denatured probe cocktail onto the denatured chromosome preparation and cover with the required sized coverslip prior to fluorescence imaging.
(18) Seal the coverslip to the slide using rubber cement.
(19) Incubate for 1–2 days in a humidified chamber at 37°C (±1°C).
(20) Following incubation, carefully remove the rubber cement and the cover slips.
(21) Place slides into the Coplin containing prewarmed (72°C (±1°C) 0.4× SSC for 2 min.
(22) Incubate slides into the Coplin containing 2× SSCT for 30 s.
(23) Briefly, wash with Milli-Q^®^ water to avoid crystal formation and leave to dry in air at room temperature.
(24) Apply 20 µl of the DAPI/antifade and overlay with a 24 × 60 mm^2^ cover slip and incubate for 10 min.
(25) Proceed with imaging and analysis. Store at –20°C for up to 2 weeks.

### Calibrations Prior to FLIM Imaging

(1) alibrate the motorized sample stage of the FLIM microscope to the epi-fluorescence microscope.
(2) To correlate chromosome images between two microscopes, use a reference glass slide “The England Finder” also known as graticules (Pyser-SGI Ltd.). The graticules is 3″ × 1″ in size, same as standard glass slides. This has a marked square grid at 1 mm intervals. Record approximately 20 x, y coordinates on the graticules using both microscopes. Generate a linear equation for both X and Y directions from the obtained coordinates and then apply the same linear equation to re-locate the chromosome spreads during the imaging with epifluorescence microscope and FLIM.
(3) This helps to locate the same chromosome spreads imaged from an inverted-multiphoton confocal to an upright-epifluorescence microscope.
(4) Measure the instrument response function (IRF) of the FLIM setup prior to any data acquisition. The reason for this is to check the excitation pulse profile of the instrument as well as correct for noise introduced into the system by for example radio-frequency pickup by the detector and electrical cables. Poorly shielded detectors may also be sources of noise. Where the noise cannot be completely eliminated, the IRF data can be used to deconvolute the noise from the data. The IRF peak should be sharp and narrow and depending on the laser pulse width and detector response, this should not be larger than the detector response if the laser width is narrower. Generally, photomultiplier tubes (PMTs) give a secondary peak in the IRF while hybrid detectors do not. It is therefore preferable to use hybrid detectors where possible. These are also less prone to damage by very high fluorescence intensities and photon count rates. Use crystals of either urea, potassium dihydrogen phosphate (KDP) or gold nanoparticles to determine the IRF when using multiphoton excitation. The NIR laser is tuned to 740–760 nm to excite the crystal followed by detection of a second harmonic signal. When using one photon excitation, a highly dilute solution of ludox may be used with the emission filters replaced by several ND filters to avoid too much laser power reaching the detector (See Section *FLIM Data Analysis* for software generated IRF modes). The use of multiphoton excitation also has several advantages over one photon excitation. For example, the use of NIR light that this less phototoxic to biological samples (in live cell experiments), reduced detector sensitivity in the red region, thus reducing background and reduced general autofluorescence. We found that the use of multiphoton excitation provided better time lifetime difference between the centromere and chromosome arm regions than using a standard 405 nm one photon excitation using light with more than 40 ps pulse-width. However, we did not investigate using 365 nm excitation where DAPI maximally absorbs. In our set up, we obtain 20 ps for a detector with a 25 ps response (Becker and Hickl HPM-100–06) or 110 ps for a detector with 110 ps response (Becker and Hickl HPM-100–40). A 3 mm BG39 band filter is used to remove the NIR laser light and transmit the fluorescence of the DAPI signal. Although photobleching does not generally affect excited state lifetime values, excessively high excitation average laser powers may lead to photo-induced fluorescent products. It is therefore best to determine any bleaching effects and to avoid higher than necessary excitation average laser powers. We used 0.1–1.0 mW, 750 nm laser power at the sample.
(5) Calibrate the setup with known fluorophores such as 10–100 µM rhodamine B, 10–100 µM erythrosin B and 10 µM fluorescein, all prepared in water, Aliquot 50 µl of the required fluorophore solution on a round glass coverslip, thickness number –1 or 1.5 (VWR international). Place on the multiphoton confocal microscope stage and raster scan using NIR light, in the dark to avoid damage to the detector. Excited fluorescence signals are detected by a hybrid detector HPM 100–40. Lifetime value of the fluorophores, 1.65, 0.12 and 4.0 ns respectively were obtained. This is within 5% error of the standard literature value and in line with our daily calibration operation values.
(6) Once an instrument test and calibration are completed, locate and record the coordinates of the slide references on the epi-fluorescence microscope on which the chromosome images have been captured (see above, *Calibrations Prior to FLIM Imaging*, subsections 1 and 2).

### FLIM Data Acquisition

**FIGURE 1:**
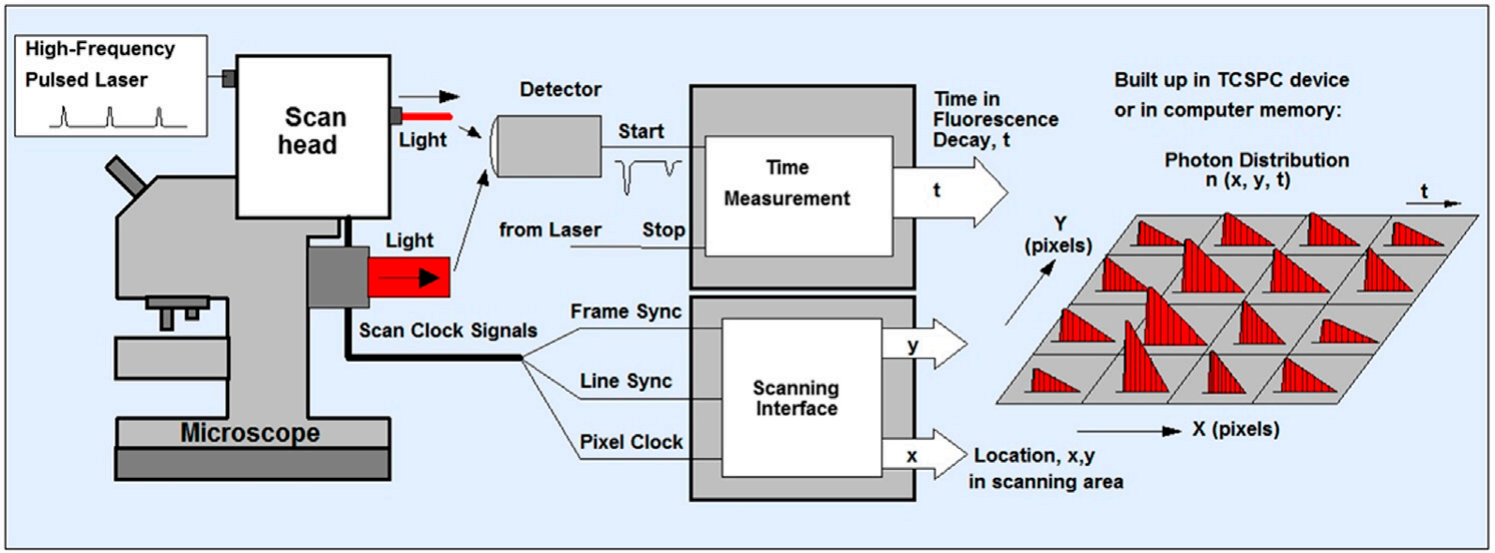
Schematics of TCSPC- FLIM. The sample is scanned using a laser scanning microscope that has a focused beam of a high-repetition-rate pulsed laser ion. The system can either be one or multiphoton depending on the laser used. The TCSPC detector is attaches to either the confocal or non-descanned port of the microscope. The detector sends an electrical pulse into the TCSPC module upon detection of every photon. The TCSPC module i) determines the decay time (t) of the photon ii) receives scan clock signals (pixel, line and frame clock) from the scan controller of the microscope and also iii) is configured as a scanning interface with two counters X, Y, for the x and y location in the scanning area. With permission from: W. Becker, The bh TCSPC handbook. 8th edition (2019) available on www.becker-hickl.com Also see (Schoberer and Botchway, 2014; Suhling et al., 2019).

(1) Prepare a slide with chromosomes, stained with 4 µM DAPI. Locate the x, y coordinates of the chromosomes using the epifluorescence microscope initially followed by the multiphoton FLIM microscope. Please note there are now FLIM instruments with combined epifluorescence attachment for chromosome identifications.
(2) Where the multiphoton microscope has a UV light (365–405 nm), it is easier to initially use a 405 nm excitation wavelength before multiphoton FLIM.
(3) Prior to multiphoton imaging mode, make sure the 405 nm laser is switched off. Due to chromatic aberration in our microscopes there is a need to adjust the focus for the 760 nm multiphoton excitation wavelength. Choose optimal excitation average power to avoid too much photobleaching of DAPI during multiphoton or confocal scanning.
(4) For TCSPC FLIM, we use the Becker and Hickl system although other systems are equally adequate such as the PicoQuant system. Using the Becker and Hickl data acquisition software SPCM (version 9.80, 64 bit), set out parameters such as number of cycles or image frames to accumulate and the pixels of the image, 512 × 512, to acquire a clear set of FLIM data (10–30 s) with each chromatid visible. Image pixel size of 128 × 128 to 2048 × 2048 may be acquired as well as lifetime decay resolution of 64–1024 (known as time bins). It is worth noting that low photon counts will determine the pixel resolution as well as the time resolution. Once the pixel number and time bins are selected, the acquisition may be operated in the scan-syn in mode where the data is stored on the TCSPC PC card, FiFo (file-in file-out) mode, where the data is stored as photon arrival time and x, y coordinates directly onto the PC memory (Figure 1).

### FLIM Data Analysis

**FIGURE 2:**
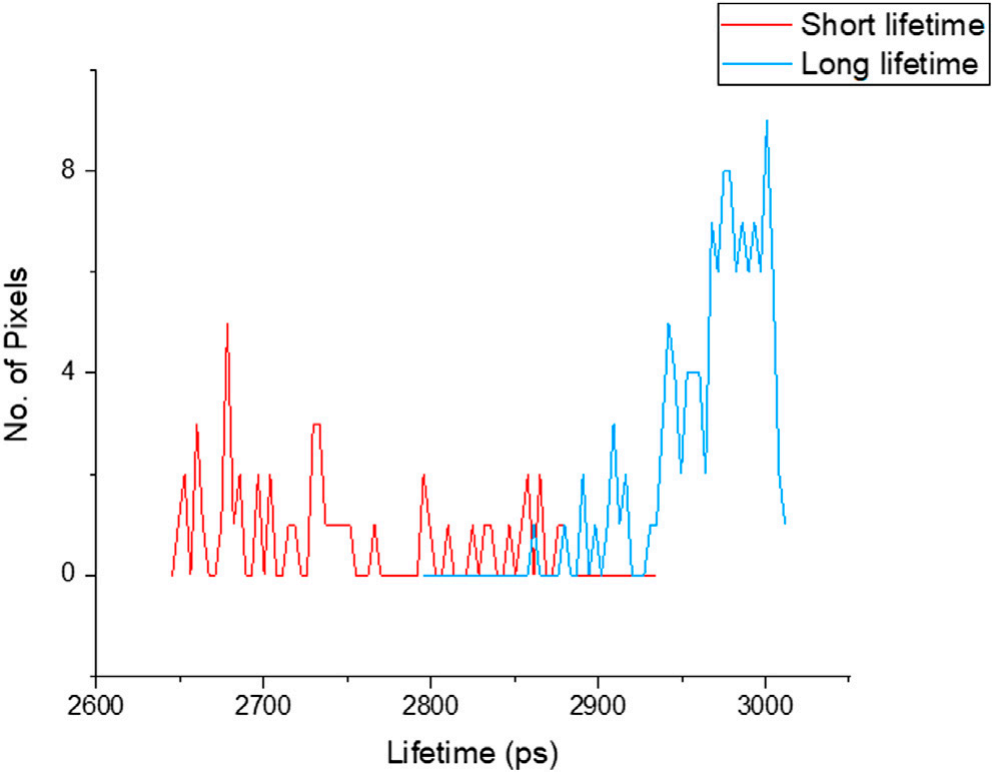
Lifetime distribution of chromosomes measured from image Figure 3, showed shorter lifetime (red 2.76 ± 0.07 ns) and longer lifetime (blue 2.95 ± 0.04 ns) values of DAPI stained chromosomes.

**FIGURE 3:**
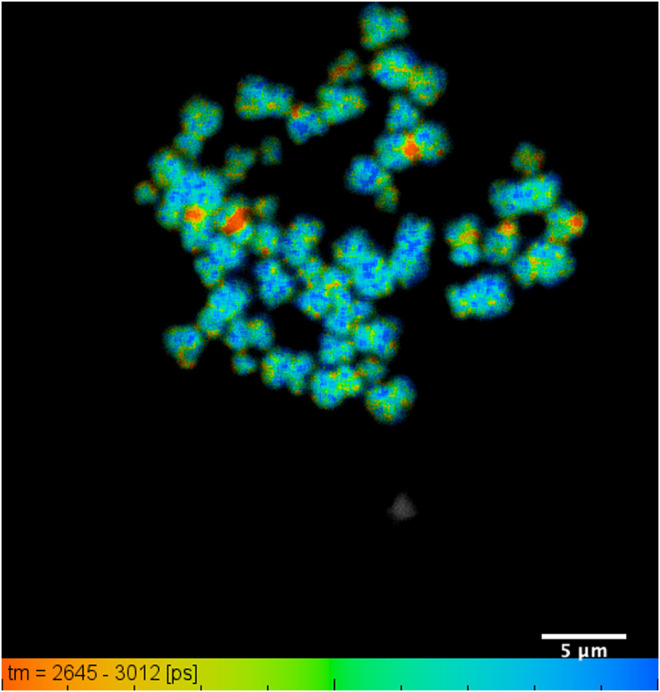
DAPI lifetime map of a typical chromosome spread with all 46 human chromosomes identified: showing lifetime change along the length of an individual chromosome, obtained from human T-lymphocytes. The lifetime values range from 2.76 ± 0.07 ns to 2.95 ± 0.04 ns within a field of view of 35 µm and scale bar of 5 µm.

**FIGURE 4:**
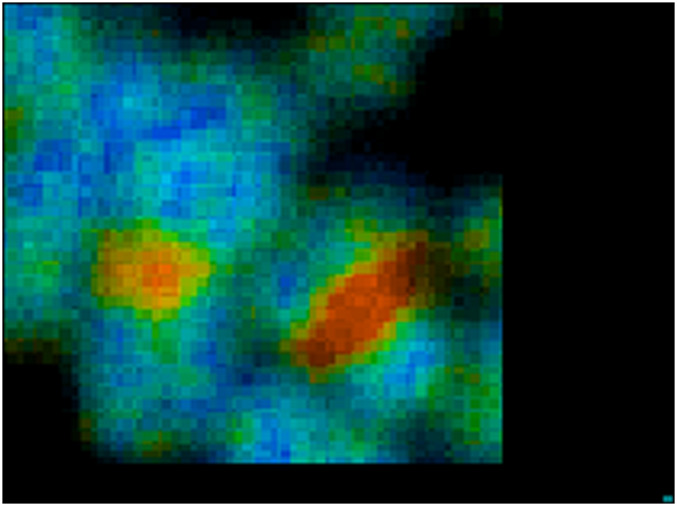
Zoomed image of chromosome 1 and 9 from image Figure 3.

**FIGURE 5:**
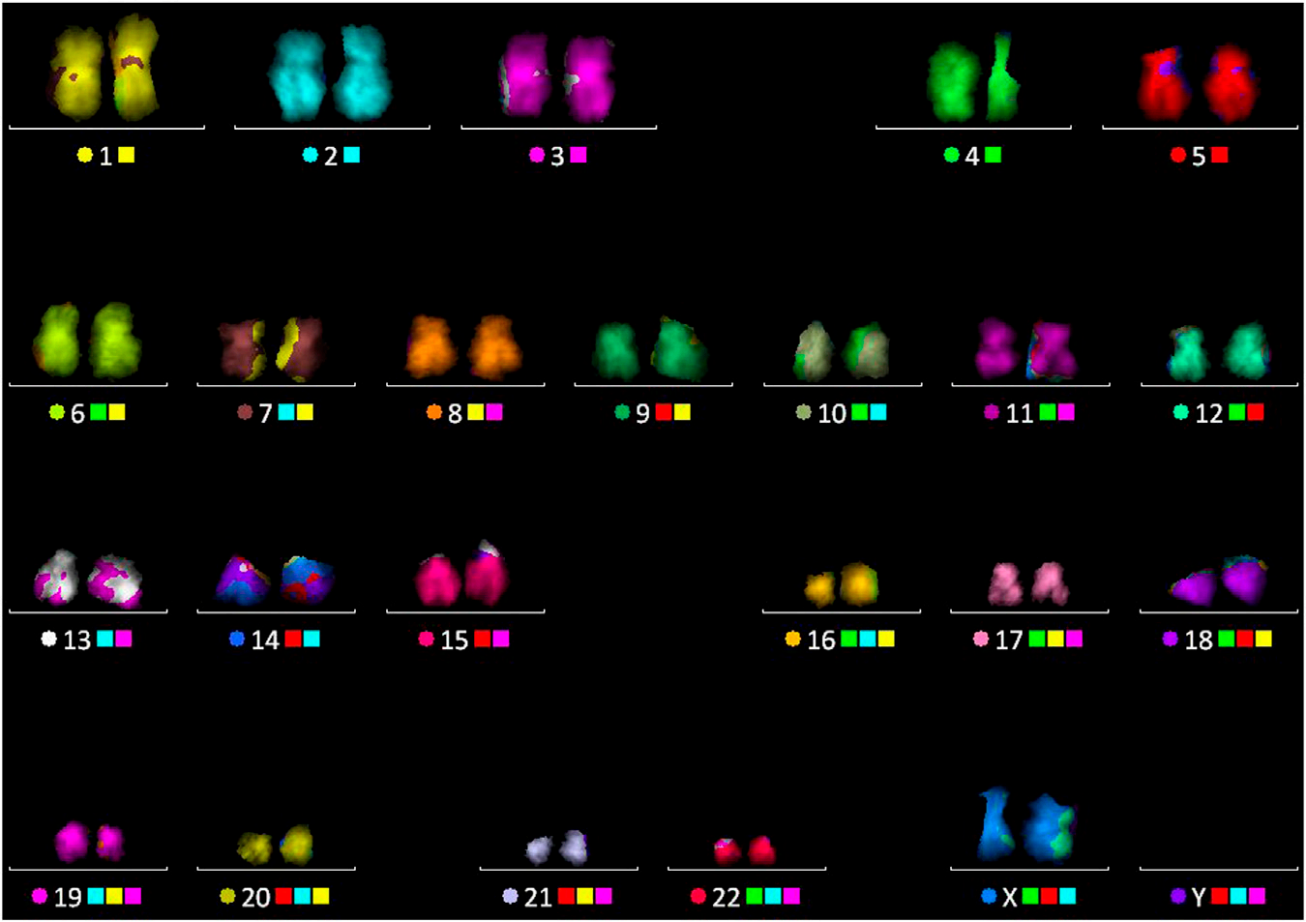
Multicolor FISH performed on the chromosome spread, after the FLIM imaging, followed by karyotype as shown in image.

(1) To analyze the raw acquired data, the data file from the TCSPC is imported into the SPCImage software (version 6.4) for processing.
(2) During FLIM data acquisition, it is often difficult to or near impossible to acquire IRF that works well for every pixel in the image. SPCImage therefore offers several options to generate an IRF. The Becker and Hickl SPCImage software uses data from an area around the brightest spot in the image to calculate an IRF. When “Auto” has been set the IRF calculation is done automatically after loading data.
(3) In the SPCImage software, it is necessary to discard pixels with poor photon signal to noise ratio and by adjusting the threshold range between 25 and 35 or allowing the software to automatically determine this. Ideally pixel binning should be kept to a minimum between 1 and 3, depending upon intensity of the image. Higher binning values likely degrade the final FLIM image.
(4) In our case we chose “Incomplete Multiexponential” decay model as our fitting model to calculate accurate fluorescence lifetime of a fluorophore (under “Option-Model-Incomplete Multiexponential”) due to our laser running at around 80 MHz (12.5 ns between laser pulses) and the lifetime of DAPI does not decay to the baseline before the next pulse.
(5) Chi-square (χ^2^) is used to determine the goodness of the decay fitting. A chi-square value of unity indicates a perfect decay curve fitting that is desirable. Chi-square (>1.4) denotes presence of multiple fluorophore components and (<0.8) represents poor fit of the data point.
(6) Set appropriate “scatter” and “shift” values then run decay matrix (under “calculate-Decay Matrix”), for whole image to obtain the lifetime distribution of whole image. BH software converts intensity image to false-colored image to generate lifetime values at each pixel.
(7) It is also necessary to set a false-color range from “Minimum” and “Maximum” (opt continuous color mode), (under “Option-Color”). This generally “spreads” out the lifetime range (example in Figure 2).
(8) Following the analysis, pseudo colored histogram denotes range of short and long lifetime of a fluorophore stained to chromosomes. Here, we consider red representing shorter lifetime and blue representing longer lifetime.
(9) The median, minimum and maximum values of the lifetime values from the distribution curve may be taken to generate the range of lifetimes per chromosome and sub-region.
At least three areas of a sample slide and at least three independent biological samples per chromosome preparation combination were analyzed, and the average of the ranges were taken.
(10) Here, FLIM measurement of the chromosome sample indicates that chromosome 1, 9, 15, 16 have a shorter lifetime at the heterochromatin regions compared to the rest of the chromosomes (Figures 3–5).

## Notes

**FIGURE 6:**
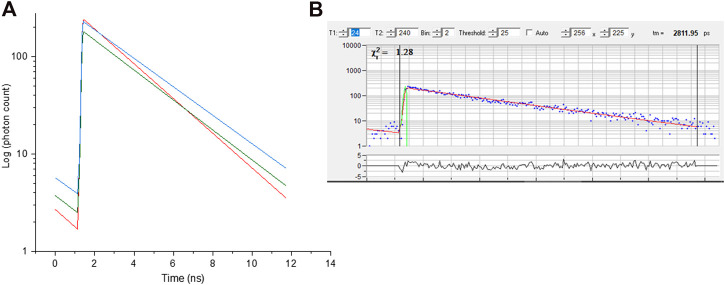
**(A)** Fluorescence decay curve obtained from selected pixel of red, green and blue regions of chromosome 9 from image Figure 3. **(B)**- representative raw decay data with a single exponential fitting.

(1) To remove unbound DAPI rinsed with milli Q water.
(2) Prepared slides were first observed under fluorescence microscope (Zeiss Z2 Axio imager with Isis software) for quality assurance of chromosome spreads then transferred to the FLIM imaging.
(3) A 10× objectives was used to locate the chromosomes and observed the quality of the prepared chromosome spreads. A 60× (water immersion) or 63× objective (with immersion oil) was used to visualize the DAPI stained chromosomes at high resolution.
(4) Store the probe away from the light during the process of denaturation.
(5) Once M-FISH cocktail is prehybridized, spin briefly to obtain probe cocktail.
(6) The fluorescence decay function (*f*) at time, *t*, is obtained from the intensities data to obtain the excited state lifetime (τ) exponential decay as follows:
  - ߦ a homogeneous population of molecules in the same environment giving a single exponential.
  - ߦ $f\left(t\right)=e^{-t/\tau }$
  - ߦ The equation for triple exponential is: $\;f\left(t\right)=ae^{-t/\tau 1}+be^{-t/\tau 2}$
  - ߦ That for a triple exponential is: $f\left(t\right)=ae^{-t/\tau 1}+be^{-t/\tau 2}+ce^{-t/\tau 3}$

the amplitudes of the exponential components, *a*, *b* and *c* define the contributions to each lifetime (example in Figure 6).

## Data Availability

The raw data supporting the conclusions of this article will be made available by the authors, without undue reservation.
